# Differential Presentations of Arterial Thromboembolic Events Between Venous Thromboembolism and Atrial Fibrillation Patients

**DOI:** 10.3389/fcvm.2021.775564

**Published:** 2021-12-06

**Authors:** Yu-Sheng Lin, Ming-Shyan Lin, Victor Chien-Chia Wu, Yung-Lung Chen, Jung-Jung Chang, Pao-Hsien Chu, Gregory Y. H. Lip, Mien-Cheng Chen

**Affiliations:** ^1^Division of Cardiology, Department of Internal Medicine, Chang Gung Memorial Hospital, Chiayi, Taiwan; ^2^College of Medicine, Graduate Institute of Clinical Medical Sciences, Chang Gung University, Taoyuan City, Taiwan; ^3^Division of Cardiology, Chang Gung Memorial Hospital, Linkou Medical Center, Taoyuan City, Taiwan; ^4^Division of Cardiology, Department of Internal Medicine, Kaohsiung Chang Gung Memorial Hospital, Chang Gung University College of Medicine, Kaohsiung, Taiwan; ^5^Liverpool Centre for Cardiovascular Science, Liverpool Heart and Chest Hospital, University of Liverpool, Liverpool, United Kingdom; ^6^Aalborg Thrombosis Research Unit, Department of Clinical Medicine, Faculty of Health, Aalborg University, Aalborg, Denmark

**Keywords:** atrial fibrillation, arterial thromboembolic event (ATE), venous thromboembolism (VTE), mortality, stroke, myocardial infarction (MI)

## Abstract

**Objective:** Atrial fibrillation (AF) and venous thromboembolism (VTE) share several risk factors related to arterial thromboembolism. No study has reported the differential contribution to arterial thromboembolic events and mortality between these two conditions in the same population. We therefore assessed the differential arterial thromboembolic events between AF and VTE.

**Methods:** We included AF and VTE national cohorts derived from Taiwan National Health Insurance Research Database between 2001 and 2013. The eligible population was 314,861 patients in the AF cohort and 41,102 patients in the VTE cohort. The primary outcome was arterial thromboembolic events, including ischemic stroke, extracranial arterial thromboembolism (ECATE) and myocardial infarction (MI). Secondary outcomes were all-cause mortality and cardiovascular death.

**Results:** After a 1:1 propensity matching, 32,688 patients in either group were analyzed. The risk of arterial thromboembolic events was lower in the VTE cohort than that in the AF cohort (subdistribution hazard ratio [SHR], 0.60; 95% confidence interval [CI], 0.57–0.62). The risk of ischemic stroke (SHR, 0.44; 95% CI, 0.42–0.46) and MI (SHR, 0.80; 95% CI, 0.72–0.89) were lower in the VTE cohort, while the risk of ECATE (SHR, 1.23; 95% CI, 1.14–1.33; particularly lower extremities) was higher in the VTE cohort. All-cause mortality rate was higher in the VTE cohort (HR, 1.18; 95% CI, 1.15–1.21) while the risk of cardiovascular death was lower in the VTE cohort (HR, 0.96; 95% CI, 0.93–0.995).

**Conclusions:** Patients with AF had higher risks of arterial thromboembolic events compared to patients with VTE, despite having risk factors in common. The VTE cohort had higher risks of all-cause mortality and ECATE, particularly lower extremity events, compared to AF patients. The differential manifestations of thromboembolism sequelae and mortality between AF and VTE patients merit further investigation.

## Introduction

Atrial fibrillation (AF) is associated with an increased risk of stroke, systemic thromboembolic events, and mortality (1). Long-term anticoagulation therapy, particularly with direct oral anticoagulants (DOACs), significantly reduces the risk of stroke and mortality (2). In terms of venous thromboembolism (VTE), including deep venous thrombosis (DVT) and pulmonary embolism (PE), the duration of anticoagulation therapy to prevent recurrences takes into consideration the risk of recurrent VTE and the risk of bleeding (3).

AF and VTE have many pathophysiological and clinical risk factors in common. In terms of pathophysiology, the pathogenesis of arterial thromboembolism in AF has been associated with a prothrombotic state by fulfilling Virchow's triad for thrombogenesis, i.e., with abnormal blood flow (stasis) in the atria, vessel wall abnormalities and abnormal blood constituents (coagulation factors) as well as inflammation (4). Likewise, the pathogenesis of arterial thromboembolism in VTE has been associated with a prothrombotic state, i.e., with abnormal blood flow (stasis) in the vessels, vessel wall abnormalities and abnormal blood constituents (coagulation factors) as well as inflammation (5, 6). Several studies showed that VTE increases risk of atherothrombotic cardiovascular events, including myocardial infarction (MI) (7). In terms of contributing factors, AF and VTE also share similar comorbidities (3, 6), such as age, hypertension, smoking, diabetes, and obesity (8–10), peripheral artery disease (11) and malignancy (12). Moreover, one community registry study reported that AF and VTE independently contributed to each other (13). However, the duration of prescribing anticoagulation is quite different between AF and VTE in current practice. Long-term anticoagulation should be prescribed for AF patients (2, 14) whereas more limited-duration of anticoagulation is sometimes prescribed for VTE patients unless there are high risk features for recurrence (3, 15).

We hypothesized that AF and VTE, despite sharing many pathophysiological and clinical risk factors, have different duration of prescribing anticoagulation and should have differential contribution to arterial thromboembolic events and mortality in the same population. Accordingly, we tested this hypothesis in a nationwide cohort study of VTE and AF patients from the Taiwan National Health Insurance Database.

## Methods

The data of this national retrospective cohort study was retrieved from the Taiwan National Health Insurance Research Database (NHIRD) released by the Taiwan National Health Research Institutes. The National Health Insurance system is a mandatory universal health insurance program that offers comprehensive medical care coverage to nearly all Taiwan residents since the inception of the program in March 1995. In the NHIRD, the patients' original identification numbers are encrypted and the encrypting procedure is consistent, so that linking claims belonging to the same enrollee is feasible and can be followed longitudinally. The available health care information included complete outpatient visits, hospitalization, and diseases, which were registered using International Classification of Diseases, Ninth Revision, Clinical Modification (ICD-9-CM) codes (16). In addition, medication prescriptions are also recorded. Patients with newly diagnosis of AF and VTE were included in this study. The study was approved by the Institutional Review Board of Chang Gung Memorial Hospital (201900915B1).

### Identification of Patients With VTE and AF

**Figure 1:**
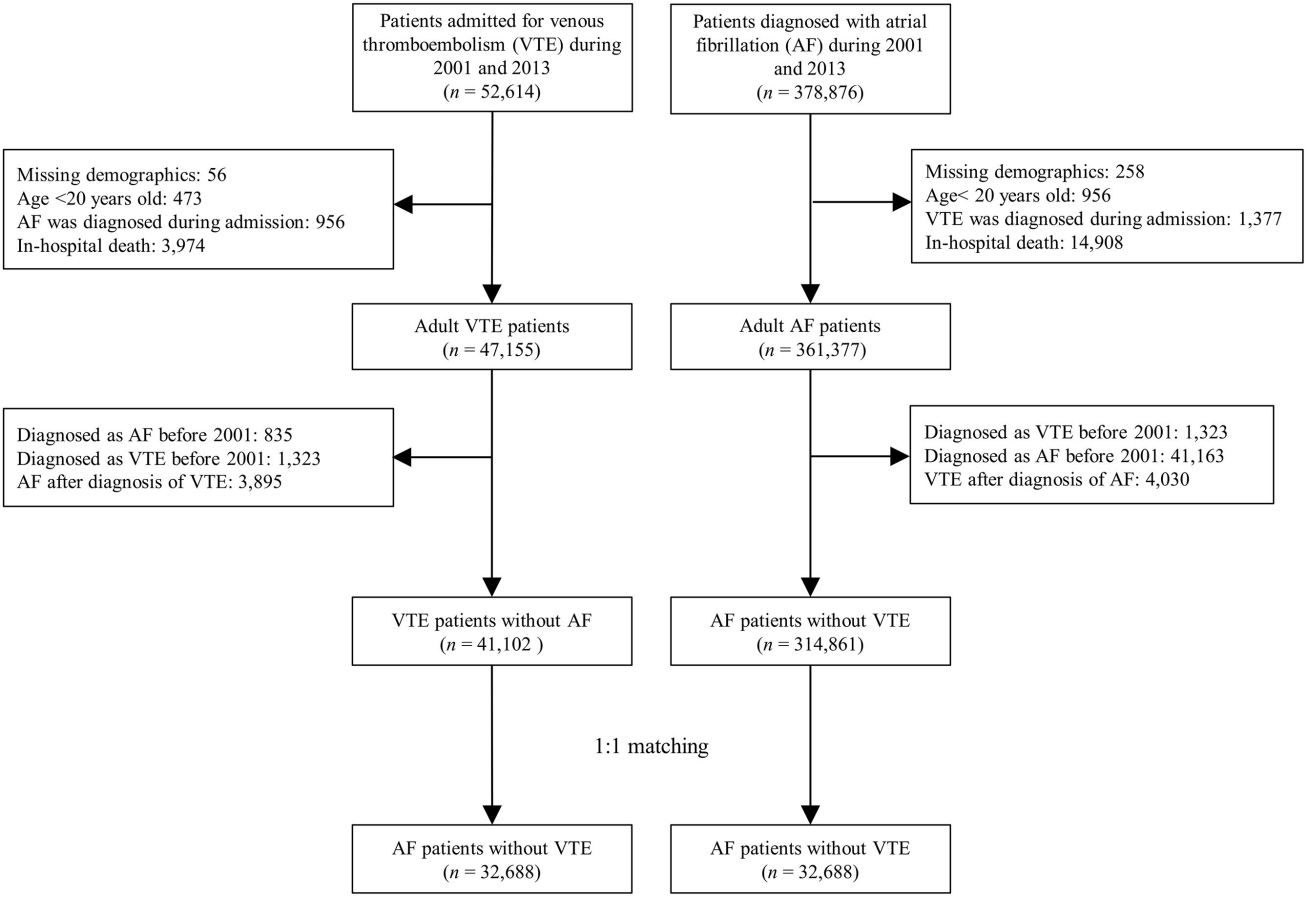
Flowchart for selection of the study patients.

This study included national AF and VTE cohorts. Patients with AF were identified with ≥2 times outpatient visits or in a discharge diagnosis using the ICD-9 CM diagnostic code of 427.31 between 2001 and 2013. Patients with VTE were identified using the discharge diagnosis (ICD-9-CM: 453 for DVT and 415.1 for PE) with use of anticoagulation during admission between 2001 and December 31, 2013. In the AF cohort, we excluded patients who were under age of 20 years old and were diagnosed as VTE historically (the diagnosis could be tracked up to year 1997) or in follow-up period. In the VTE cohort, we excluded patients who were under age of 20 years old and were diagnosed as AF historically (the diagnosis could be tracked up to year 1997) or in the follow-up period. In order to compare the differences in the clinical outcomes after developing AF and VTE, we excluded those who died at the index admission in both cohorts. Finally, 314,861 AF patients without VTE and 41,102 VTE patients without AF were included in this study (Figure 1).

### Covariates

Covariates were age, sex, eighteen comorbidities, Charlson Comorbidity Index score, four historical events, and fourteen kinds of medications (Table 1). Comorbidities were recognized with at least two clinic visits or any inpatient record in the previous year before the index date. Historical events were detected using any inpatient diagnosis before the index date which could be tracked up to year 1997. The use of medication was extracted within 3 months after index date. All the information about medications were extracted from the claims data of outpatient visits or the refill for chronic illness in the pharmacy by using the Anatomical Therapeutic Chemical codes or the Taiwan NHI reimbursement code.

**Table 1:** Baseline characteristics of the patients diagnosed with AF or VTE.

	**Before propensity matching**			**After propensity matching**		
	**VTE** **(*n* = 41,102)**	**AF** **(*n* = 314,861)**	**STD**	**VTE** **(*n* = 32,688)**	**AF** **(*n* = 32,688)**	**STD**
Age (years)	64.3 ± 16.4	71.2 ± 13.5	−0.46	66.7 ± 15.4	67.4 ± 14.6	−0.04
**Age group**						
<65 years	18,837 (45.8)	89,486 (28.4)	0.37	13,087 (40.0)	12,464 (38.1)	0.04
65-74 years	9,526 (23.2)	82,459 (26.2)	−0.07	8,082 (24.7)	8,422 (25.8)	−0.02
≥ 75 years	12,739 (31.0)	142,916 (45.4)	−0.30	11,519 (35.2)	11,802 (36.1)	−0.02
Male sex	19,152 (46.6)	174,954 (55.6)	−0.18	16,123 (49.3)	16,752 (51.2)	−0.04
**Types of VTE**						
Pulmonary embolism (PE)	9,143 (22.2)	-		7,702 (23.6)	-	
Deep vein thrombosis (DVT)	29,512 (71.8)	-		23,081 (70.6)	-	
DVT + PE	2,447 (6.0)	-		1,905 (5.8)	-	
**Comorbid conditions**						
Hypertension	21,262 (51.7)	191,993 (61.0)	−0.19	18,355 (56.2)	18,942 (57.9)	−0.04
Diabetes mellitus	10,800 (26.3)	75,303 (23.9)	0.05	8,877 (27.2)	8,880 (27.2)	<0.01
Ischemic heart disease	8,845 (21.5)	110,828 (35.2)	−0.31	8,023 (24.5)	8,695 (26.6)	−0.05
Dyslipidemia	7,382 (18.0)	52,525 (16.7)	0.03	6,133 (18.8)	6,423 (19.6)	−0.02
Gout	4,104 (10.0)	31,540 (10.0)	<0.01	3,463 (10.6)	3,526 (10.8)	−0.01
COPD	5,630 (13.7)	58,210 (18.5)	−0.13	4,843 (14.8)	4,793 (14.7)	<0.01
Peripheral artery disease	2,802 (6.8)	11,400 (3.6)	0.14	2,052 (6.3)	2,041 (6.2)	<0.01
Chronic kidney disease	7,190 (17.5)	45,300 (14.4)	0.08	5,803 (17.8)	5,758 (17.6)	<0.01
Dialysis	1,209 (2.9)	8,190 (2.6)	0.02	1,034 (3.2)	1,065 (3.3)	−0.01
Cancer	8,847 (21.5)	19,901 (6.3)	0.45	5,030 (15.4)	4,727 (14.5)	0.03
Auto-immune disease	811 (2.0)	1,891 (0.6)	0.12	424 (1.3)	393 (1.2)	0.01
Hepatitis C virus infection	835 (2.0)	4,841 (1.5)	0.04	626 (1.9)	593 (1.8)	0.01
Paralysis	3,245 (7.9)	22,491 (7.1)	0.03	2,705 (8.3)	2,810 (8.6)	−0.01
Osteoporosis	3,578 (8.7)	19,662 (6.2)	0.09	2,702 (8.3)	2,503 (7.7)	0.02
Charlson comorbidity index score	3.0 ± 3.0	2.0 ± 2.0	0.40	2.7 ± 2.7	2.7 ± 2.7	<0.01
**History of disease**						
Prior any stroke	5,600 (13.6)	46,969 (14.9)	−0.04	4,933 (15.1)	5,230 (16.0)	−0.03
Prior ischemic stroke or systemic thromboembolism	5,506 (13.4)	45,757 (14.5)	−0.03	4,832 (14.8)	5,228 (16.0)	−0.03
Old MI	1,249 (3.0)	13,564 (4.3)	−0.07	1,140 (3.5)	1,240 (3.8)	−0.02
Heart failure admission	3,654 (8.9)	40,121 (12.7)	−0.12	3,331 (10.2)	3,608 (11.0)	−0.03
**Antithrombotic therapy within 3 months after index date**						
None	9,640 (23.5)	119,550 (38.0)	−0.32	9,475 (29.0)	9,012 (27.6)	0.03
Antiplatelet	3,192 (7.8)	148,174 (47.1)	−0.98	3,189 (9.8)	3,364 (10.3)	−0.02
Anticoagulant	28,270 (68.8)	47,137 (15.0)	1.30	20,024 (61.3)	20,312 (62.1)	−0.02
**Medication**						
ACEi or ARB	10,771 (26.2)	137,957 (43.8)	−0.38	9,956 (30.5)	11,094 (33.9)	−0.07
Beta blocker	8,044 (19.6)	115,262 (36.6)	−0.39	7,496 (22.9)	8,337 (25.5)	−0.06
DCCB	9,594 (23.3)	85,295 (27.1)	−0.09	8,193 (25.1)	8,447 (25.8)	−0.02
Diuretic	9,849 (24.0)	80,723 (25.6)	−0.04	7,870 (24.1)	8,237 (25.2)	−0.03
Metformin	4,078 (9.9)	35,237 (11.2)	−0.04	3,428 (10.5)	3,540 (10.8)	−0.01
TZD	656 (1.6)	4,942 (1.6)	<0.01	520 (1.6)	506 (1.5)	<0.01
DPP4i	774 (1.9)	5,783 (1.8)	<0.01	681 (2.1)	742 (2.3)	−0.01
Insulin	1,900 (4.6)	10,125 (3.2)	0.07	1,476 (4.5)	1,458 (4.5)	<0.01
Estrogen	946 (2.3)	4,105 (1.3)	0.08	577 (1.8)	504 (1.5)	0.02
Antidepressants	3,728 (9.1)	20,564 (6.5)	0.09	2,754 (8.4)	2,747 (8.4)	<0.01
Statin	4,289 (10.4)	39,303 (12.5)	−0.06	3,846 (11.8)	4,264 (13.0)	−0.04
Digoxin	1,068 (2.6)	73,147 (23.2)	−0.65	1,067 (3.3)	1,257 (3.8)	−0.03
Follow up year	3.8 ± 3.5	4.2 ± 3.4	−0.12	3.7 ± 3.4	3.8 ± 3.4	−0.03

*VTE, venous thromboembolism; AF, atrial fibrillation; STD, standardized difference; COPD, chronic obstructive pulmonary disease; ACEi, angiotensin converting enzyme inhibitor; ARB, angiotensin receptor blocker; DCCB, dihydropyrinde calcium channel blocker; TZD, thiazolidinedione; DDP4i, dipeptidyl peptidase 4 inhibitor*.

*Data were presented as frequency (percentage) or mean ± standard deviation*.

### Outcomes

The primary outcome was arterial thromboembolic events, including ischemic stroke, myocardial infarction (MI) and extracranial arterial thromboembolism (ECATE). Extracranial arterial thromboembolism included arterial thromboembolic occlusion of an extremity or extracranial vital organ, including kidney, intestine, and spleen. The occurrence of ischemic stroke and MI was defined as the principal discharge diagnosis of hospitalization. The occurrence of ECATE was defined as the principal or secondary diagnoses of hospitalization. Secondary outcomes were all-cause mortality and cardiovascular death. All-cause mortality was defined as withdrawal from the NHI program (17). The definition of cardiovascular (CV) death was the criteria of the Standardized Definitions for Cardiovascular and Stroke Endpoint Events in Clinical Trials by the FDA in the United States. Each patient was followed from the discharge date of index admission to the date of event occurrence, date of death, or December 31, 2013.

### Ascertainment of VTE, AF, Ischemic Stroke, MI, and ECATE

The validation of AF has been assessed and presented in our previous reports, with a high positive predictive value (PPV) of 89% (18). The accuracy of VTE was reliable in the Taiwan insurance claim system and some published studies also used the same diagnosis method (11, 19). Ischemic stroke and MI were also validated with high PPVs (20, 21). In terms of ECATE, a validation study was conducted at our medical center, randomly sampling 100 hospitalizations for systemic thromboembolism who were selected using the same criteria as mentioned in this study. After experienced physicians (YSL and VCCW) reviewed the medical records and all imaging results, including vascular duplex, computed tomography angiography and intervention reports, the PPV of systemic thromboembolism was 88% (data not shown).

### Statistics

There would be substantial difference in the baseline characteristics between study groups (i.e., VTE and AF cohorts). Therefore, we performed 1:1 ratio propensity score matching to make the covariates balanced between groups. The propensity score was the predicted probability to be in the one group (i.e., VTE) given the values of covariates using the multivariable logistic regression without considering interaction effects. The variables selected to calculate propensity score were listed in Table 1 where the follow-up year was replaced with the index date (Table 1). The matching was processed using a greedy nearest neighbor algorithm with a caliper of 0.2 times of the standard deviation of the logit of propensity score, with random matching order and without replacement. The quality of matching was checked using the absolute value of standardized difference (STD) between the groups, where a value <0.1 was considered negligible difference. We additionally performed three propensity score matchings to compare the PE-only cohort with the DVT-only cohort, the DVT-only cohort with the AF cohort and the PE-only cohort with the AF cohort, respectively.

As to the time to fatal outcomes (i.e., all-cause mortality and cardiovascular death), the risks between the groups were compared by the Cox proportional hazard model. The incidences of time to non-fatal outcomes (e.g., ischemic stroke or MI) between groups were compared by the Fine and Gray subdistribution hazard model which considered all-cause mortality a competing risk. The within-pair clustering of outcomes after propensity score matching was accounted for by using a robust standard error (22). Finally, we performed a subgroup analysis stratified by the use of oral anticoagulant within 3 months after the index date. A two-sided *P*-value < 0.05 was considered statistically significant. Statistical analyses were performed using SAS version 9.4 (SAS Institute, Cary, NC).

## Results

### Baseline Characteristics

This study enrolled 314,861 AF patients (mean age of 71.2 ± 13.5 years) and 41,102 VTE patients (mean age of 64.3 ± 16.4 years). These two cohorts were different in age distribution, whereby the VTE cohort was predominantly age < 65 years while the AF cohort was predominantly age ≥ 75 years (Table 1). The AF cohort had significantly greater prevalence of hypertension, ischemic heart disease, chronic obstructive pulmonary disease (COPD), hyperthyroidism and heart failure, while VTE cohort had higher prevalence of peripheral artery disease, cancer and auto-immune disease. In terms of medications, angiotensin converting enzyme inhibitors/angiotensin receptor blockers, beta blockers and digoxin were more frequently prescribed in the AF population. In this study, anticoagulant use was continuous in the AF cohorts while the duration of anticoagulant was at least for 3~6 months in the VTE cohort and 38.6% of VTE patients had the duration of anticoagulant use for more than 6 months. In addition, the available follow-up period in AF cohort were longer than that in VTE cohort (AF cohort vs. VTE cohort: 4.2 ± 3.4 vs. 3.8 ± 3.5 years, standardized difference: −0.12).

After matching, 32,688 patients in either cohort were well-balanced in baseline characteristics, including follow-up period (Table 1). In patients with DVT-only vs. AF, the AF cohorts had higher incidence of ischemic heart disease, heart failure and COPD while DVT-only cohort had higher incidence of cancer, auto-immune disease and peripheral artery disease (Supplementary Table 1). In patients with PE-only vs. AF, the PE-only cohort had a higher incidence of cancer (Supplementary Table 2). In terms of the PE-only vs. DVT-only cohorts, the PE-only cohort had higher incidence of ischemic heart disease, heart failure and COPD while DVT-only cohort had higher prevalence of cancer and peripheral artery disease (Supplementary Table 3).

### Outcomes Between VTE and AF Cohorts

**Figure 2:**
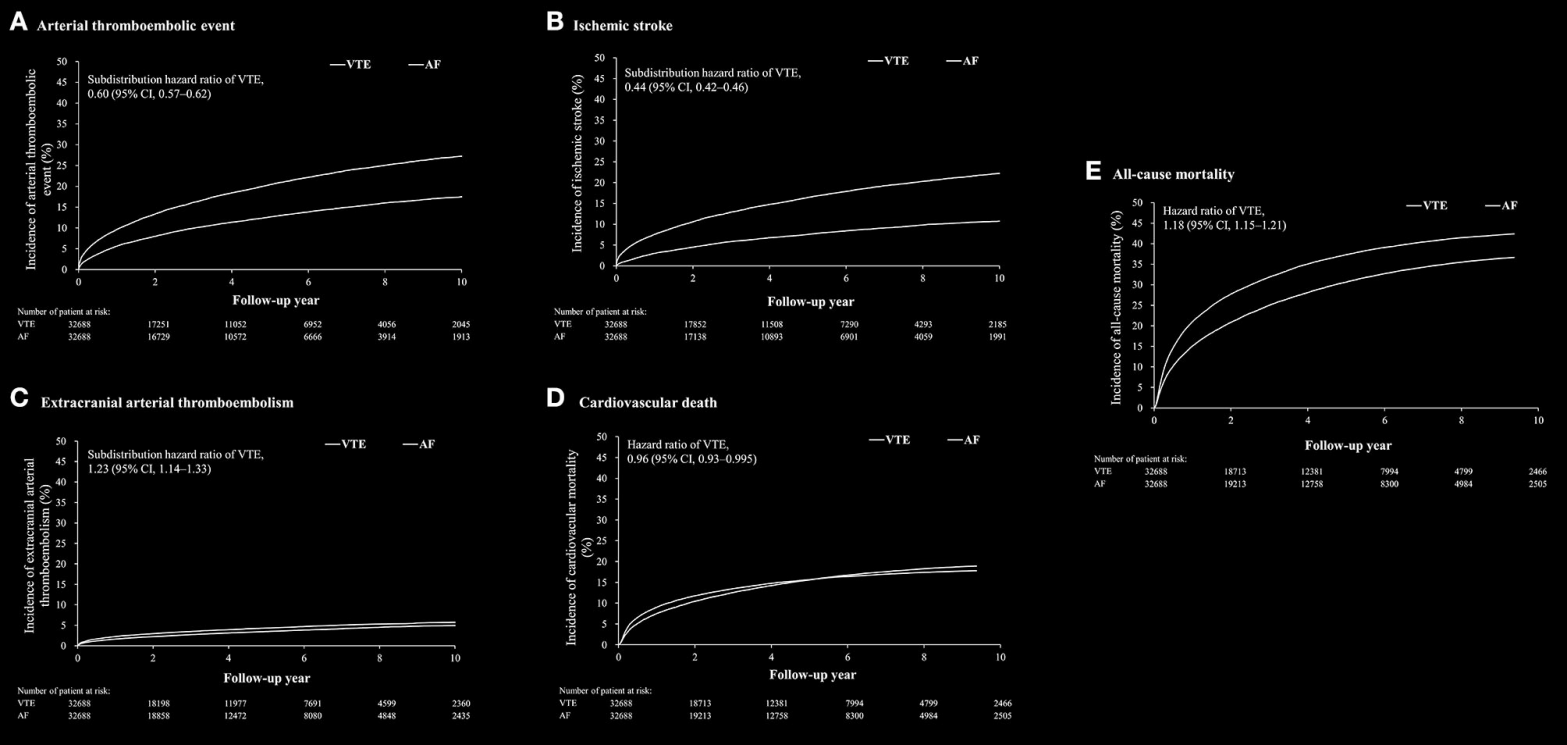
The cumulative incidence rate of arterial thromboembolic event **(A)**, ischemic stroke **(B)**, extracranial arterial thromboembolic events **(C)**, cardiovascular death **(D)** and all-cause mortality **(E)** after propensity score matching between patients with venous thromboembolism (VTE) and patients with atrial fibrillation.

The outcomes between VTE and AF cohort obtained after propensity matching are shown in Table 2; Figure 2. The risk of the arterial thromboembolic events was lower in the VTE cohort [subdistribution hazard ratio (SHR), 0.60; 95% confidence interval (CI), 0.57–0.62] (Figure 2A), as were the risks of ischemic stroke (SHR, 0.44; 95% CI, 0.42–0.46) (Figure 2B) and MI (SHR, 0.80; 95% CI, 0.72–0.89). The risks of ECATE (SHR, 1.23; 95% CI, 1.14–1.33) (Figure 2C) and all-cause mortality rate (HR, 1.18; 95% CI, 1.15–1.21) were higher in VTE cohort (Figure 2E), although the latter had lower CV death (HR, 0.96; 95% CI, 0.93–0.995) (Figure 2D).

**Table 2:** Follow-up outcomes in patients with VTE versus those with AF.

	**Before propensity matching**		**After propensity matching**			
**Outcome**	**VTE** **(*n* = 41,102)**	**AF** **(*n* = 314,861)**	**VTE** **(*n* = 32,688)**	**AF** **(*n* = 32,688)**	**HR or SHR of VTE** **(95% CI)**	***P***
Arterial thromboembolic events	4,864 (11.8)	61,684 (19.6)	4,143 (12.7)	6,383 (19.5)	0.60 (0.57–0.62)	<0.001
Ischemic stroke	2,881 (7.0)	47,867 (15.2)	2,481 (7.6)	5,141 (15.7)	0.44 (0.42–0.46)	<0.001
Extracranial arterial thromboembolism	1,725 (4.2)	10,026 (3.2)	1,410 (4.3)	1,122 (3.4)	1.23 (1.14–1.33)	<0.001
Lower extremity thromboembolism	1,435 (3.5)	8,361 (2.7)	1,184 (3.6)	917 (2.8)	1.26 (1.16–1.37)	<0.001
Non-lower extremity thromboembolism	365 (0.9)	2,329 (0.7)	288 (0.88)	284 (0.87)	0.99 (0.84–1.16)	0.867
Myocardial infarction	690 (1.7)	9,718 (3.1)	619 (1.9)	741 (2.3)	0.80 (0.72–0.89)	<0.001
Secondary outcomes						
All-cause mortality	18,098 (44.0)	135,551 (43.1)	14,188 (43.4)	12,345 (37.8)	1.18 (1.15–1.21)	<0.001
Cardiovascular death	7,321 (17.8)	73,344(23.3)	5,958 (18.2)	6,376 (19.5)	0.96 (0.93–0.995)	0.025
Non-cardiovascular death	10,777 (26.2)	62,207 (19.8)	8,230 (25.2)	5,969 (18.3)	1.42 (1.37–1.47)	<0.001

*VTE, venous thromboembolism; AF, atrial fibrillation; HR, hazard ratio; SHR, subdistribution hazard ratio; CI, confidence interval*.

*Data were presented as frequency (percentage)*.

In subgroup analysis of the cause of death after propensity matching, the percentage of CV death was significant higher in AF cohort than in VTE cohort (VTE cohort vs. AF cohort: 56.4 vs. 66.6%, *P* < 0.001) (Supplementary Table 4). The percentages of death related to cancer and infection other than pneumonia among the causes of non-CV death were significantly higher in the VTE cohort than in the AF cohort.

Furthermore, the outcomes between the subgroups stratified according to the use of anticoagulation therapy within 3 months after index date were analyzed. The impact of anticoagulant use on the association between AF/VTE and the risk of arterial thromboembolic events was very significant (non-anticoagulant user: SHR, 0.89; 95% CI, 0.83–0.96; anticoagulant user: SHR, 0.49; 95% CI, 0.47–0.52; *P* interaction < 0.001). In terms of individual arterial thromboembolic events and mortality, anticoagulant use also had a significant impact on the association between AF/VTE and these events, except for MI (Table 3).

**Table 3:** Subgroup analysis of long-term outcomes of the VTE versus AF patients stratified by use of anticoagulation therapy within 3 months after index date in the propensity score matched cohort.

**Outcome/ Subgroup**	**VTE** **(*n* = 32,688)**	**AF** **(*n* = 32,688)**	**HR or SHR of VTE (95% CI)**	***P* for interaction**
**Arterial thromboembolic events**				<0.001
Non-anticoagulant user	1,422 (11.2)	1,583 (12.8)	0.89 (0.83–0.96)	
Anticoagulant user	2,721 (13.6)	4,800 (23.6)	0.49 (0.47–0.52)	
**Ischemic stroke**				<0.001
Non-anticoagulant user	822 (6.5)	1,141 (9.2)	0.82 (0.75–0.89)	
Anticoagulant user	1,659 (8.3)	4,000 (19.7)	0.37 (0.35–0.39)	
**Extracranial arterial thromboembolism**				<0.001
Non-anticoagulant user	497 (3.9)	328 (2.7)	1.53 (1.33–1.75)	
Anticoagulant user	913 (4.6)	794 (3.9)	1.10 (1.001–1.21)	
**Lower extremity thromboembolism**				<0.001
Non-anticoagulant user	422 (3.3)	272 (2.2)	1.56 (1.34–1.82)	
Anticoagulant user	762 (3.8)	645 (3.2)	1.13 (1.02–1.26)	
**Non-lower extremity thromboembolism**				0.016
Non-anticoagulant user	96 (0.8)	71 (0.6)	1.35 (0.99–1.84)	
Anticoagulant user	192 (1.0)	213 (1.0)	0.86 (0.71–1.05)	
**Myocardial infarction**				0.349
Non-anticoagulant user	231 (1.8)	269 (2.2)	0.86 (0.72–1.02)	
Anticoagulant user	388 (1.9)	472 (2.3)	0.77 (0.68–0.88)	
**All-cause mortality**				<0.001
Non-anticoagulant user	7,519 (59.4)	6,324 (51.1)	1.37 (1.32–1.42)	
Anticoagulant user	6,669 (33.3)	6,021 (29.6)	1.07 (1.04–1.11)	
**Cardiovascular death**				<0.001
Non-anticoagulant user	2,676 (21.1)	2,619 (21.2)	1.19 (1.12–1.25)	
Anticoagulant user	2,769 (13.8)	3,301 (16.3)	0.81 (0.77–0.85)	
**Non-cardiovascular death**				0.020
Non-anticoagulant user	4,843 (38.2)	3,705 (29.9)	1.50 (1.44–1.57)	
Anticoagulant user	3,900 (19.5)	2,720 (13.4)	1.39 (1.33–1.46)	

*VTE, venous thromboembolism; AF, atrial fibrillation; HR, hazard ratio; SHR, subdistribution hazard ratio; CI, confidence interval*.

### Outcomes Between DVT-Only and AF Cohorts

After propensity matching, there was no substantial difference in the baseline characteristics between the DVT-only and AF cohorts (Supplementary Table 1). Clinical outcomes between DVT-only and AF cohorts are shown in Supplementary Table 5.

The incidence of arterial thromboembolic event was lower in the DVT-only cohort than that in the AF cohort (SHR, 0.62; 95% CI, 0.59–0.65) (Supplementary Figure 1A). The risks of ischemic stroke (SHR, 0.45; 95% CI, 0.43–0.48) (Supplementary Figure 1B) and MI (SHR, 0.76; 95% CI, 0.67–0.86) were lower, while the risk of ECATE was higher, in the DVT-only cohort (SHR, 1.31; 95% CI, 1.20–1.43) (Supplementary Figure 1C). All-cause mortality rates were higher in the DVT-only cohort (HR, 1.14; 95% CI, 1.11–1.17), while the rate of CV death was lower (HR, 0.89; 95% CI, 0.85–0.92) (Supplementary Figures 1D,E).

### Outcomes Between PE-Only and AF Cohorts

After propensity matching, there was no substantial difference in the baseline characteristics between the PE alone and AF cohorts (Supplementary Table 2). Clinical outcomes between PE-only and AF cohorts were shown in Supplementary Table 6. The incidence of the arterial thromboembolic event was lower in the PE-only cohort than that in AF cohort (SHR, 0.52; 95% CI, 0.48–0.56) (Supplementary Figure 2A). The risk of ischemic stroke was lower in the PE-only cohort (SHR, 0.41; 95% CI, 0.37–0.45) (Supplementary Figure 2B) with no differences in ECATE (Supplementary Figure 2C) and MI event rates (Supplementary Table 6). The PE-only cohort had higher all-cause mortality (HR, 1.26; 95% CI, 1.20–1.32) and CV death (HR, 1.14; 95% CI, 1.07–1.22) than the AF cohort (Supplementary Figures 2D,E).

### Outcomes Between PE-Only and DVT-Only Cohorts

**Figure 3:**
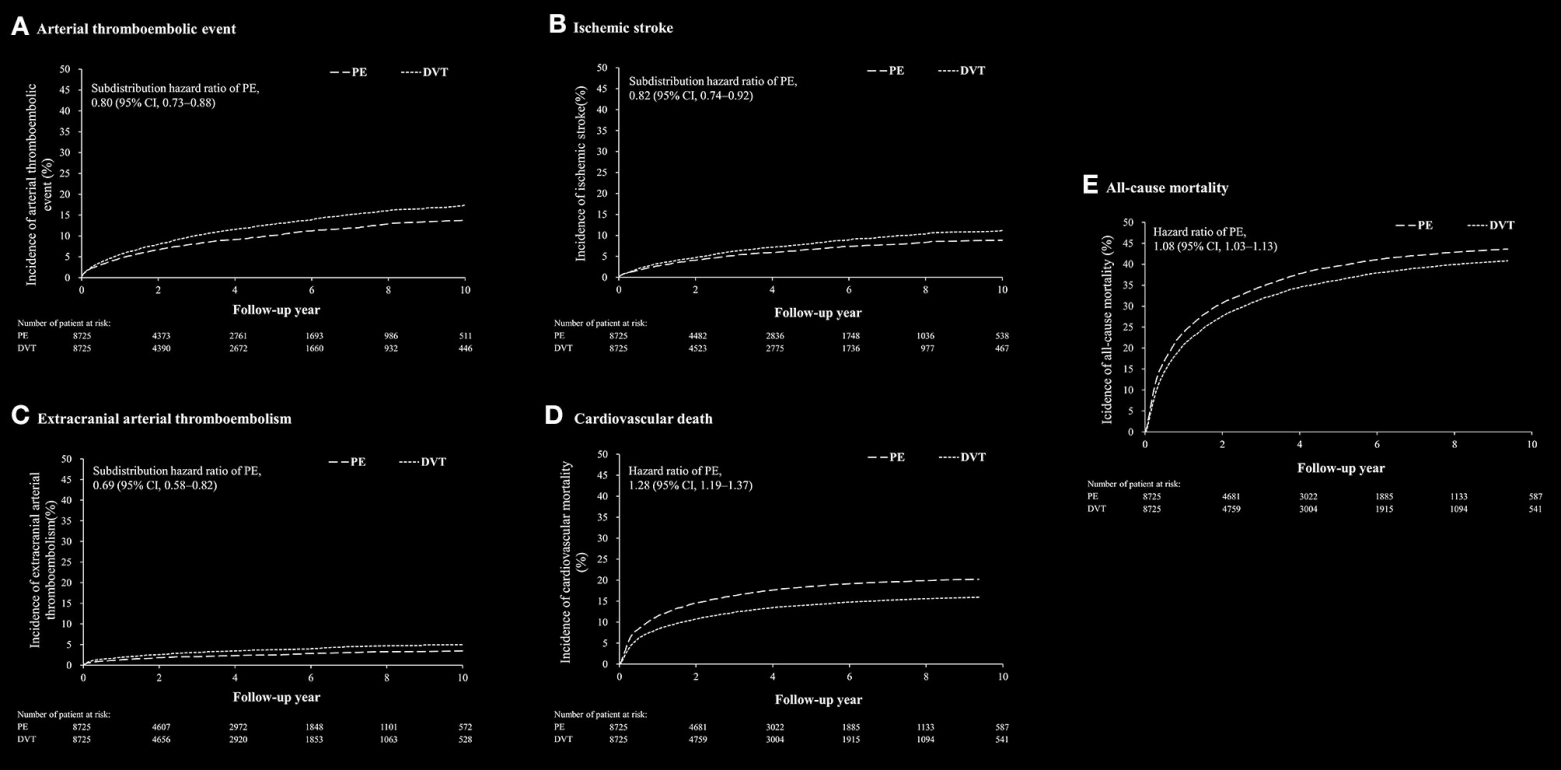
The cumulative incidence rate of arterial thromboembolic event **(A)**, ischemic stroke **(B)**, extracranial arterial thromboembolic events **(C)**, cardiovascular death **(D)** and all-cause mortality **(E)** after propensity score matching between patients with pulmonary embolism (PE) alone and patients with deep vein thrombosis (DVT) alone.

After propensity matching, there was no substantial difference in the baseline characteristics between the PE-only and DVT-only cohorts (Supplementary Table 3). Clinical outcomes between PE-only and DVT-only cohorts were shown in Supplementary Table 7. The arterial thromboembolic event was lower in the PE-only cohort than in the DVT-only cohort (SHR, 0.80; 95% CI, 0.73–0.88) (Figure 3A). The risks of ischemic stroke (SHR, 0.82; 95% CI, 0.74–0.92) and ECATE, including lower extremity events, (SHR, 0.69; 95% CI, 0.58–0.82) were lower in the PE-only cohort than in the DVT-only cohort (Figures 3B,C). The risks of all-cause mortality (HR, 1.08; 95% CI, 1.03–1.13) and CV death (HR, 1.28; 95% CI, 1.19–1.37) were higher in the PE-only cohort compared to the DVT-only cohort (Figures 3D,E).

## Discussion

**Figure 4:**
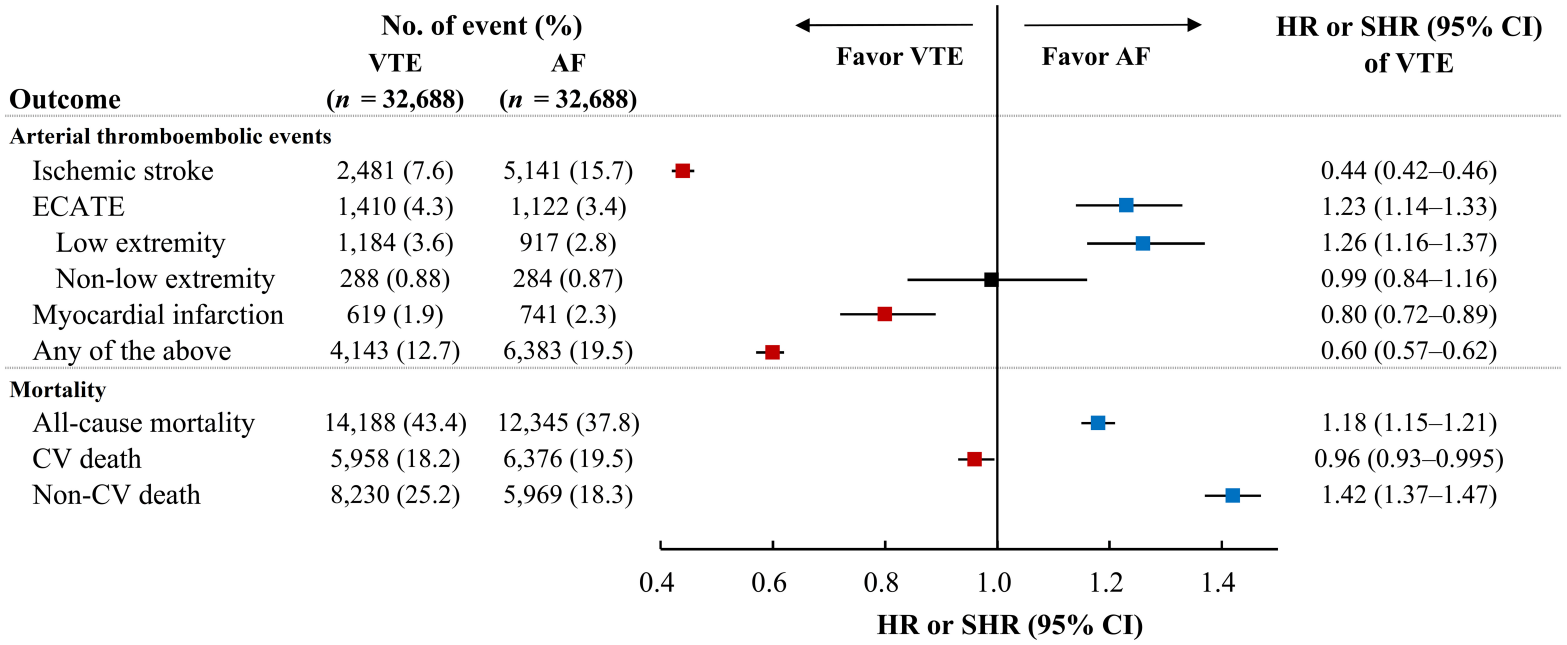
The incidence of arterial thromboembolic events and mortality between VTE and AF cohorts. The incidence of ischemic stroke and myocardial infarction were lower in VTE cohort than AF cohort but ECATE, particularly in low extremity, was lower in AF cohort than VTE cohort. In terms of mortality, CV death was lower in VTE cohorts than AF cohort while all-cause mortality and non-CV death were lower in AF cohort than VTE cohort. AF, atrial fibrillation; CV, cardiovascular; ECATE, extracranial arterial thromboembolic event; VTE, venous thromboembolism.

This retrospective 10-year nationwide cohort study enrolled two national cohorts shows that the arterial thromboembolic events, ischemic stroke and MI, were higher in matched patients with AF cohort than those with VTE cohort. Second, the VTE cohort had higher incidence of ECATE than AF cohort, particularly lower extremity thromboembolism. Third, the AF cohort had higher incidence of CV death, but lower incidence of all-cause mortality compared to the VTE cohort (Figure 4; Supplementary Table 8). In subgroup analyses comparing the DVT-only, PE-only and AF cohorts, the AF patients had highest incidence of ischemic stroke among the three cohorts and had similar incidence of MI compared to patients with PE-only. Patients with DVT-only had highest incidence of ECATE among the three cohorts, particularly lower extremity thromboembolic event. In terms of mortality, patients with PE-only had highest incidence of CV death and all-cause mortality (Supplementary Table 8).

A national 20-year observational study demonstrated that patients with VTE had a 1.26-1.31-fold increased risk of subsequent arterial thromboembolic events, including MI and stroke (23). Schulman et al. also showed that VTE was associated with a 1.28-fold increased risk of MI or stroke over a 10-year follow-up period (24). Epidemiological studies and meta-analysis have also recognized that AF is independently associated with a five-fold increased risk of stroke (1), 1.47-fold increased risk of MI (25), and a two-fold increased risk of mortality (26).

Although VTE and AF contribute to similar arterial thromboembolic events, we are unaware of any study that has compared the different presentations of arterial thromboembolic events between VTE and AF patients, from the same population cohort. Based on our study, AF contributed to more arterial thromboembolic events while VTE contributed to greater all-cause mortality. In terms of the causes of mortality, more AF patients (66.6%) died from cardiovascular death than VTE patients (56.4%) while more VTE patients (13.7%) died from cancer than AF patients (8.6%) (Supplementary Table 4). Our results were generally consistent with other studies in terms of causes of death in VTE and AF patients (27).

As mentioned above there are many parallels between the epidemiology of risk factors and associated pathogenesis of thrombosis in AF and VTE (4, 6, 8, 10, 28). Nonetheless, our study showed some differential distributions of baseline characteristics between VTE and AF cohorts. Of note, the prevalence of peripheral artery disease and cancer was higher in VTE cohort than AF cohort. In contrast, the prevalence of ischemic heart disease and heart failure was higher in AF cohort than VTE cohort. Importantly, even after propensity matching, AF cohort had higher risks of arterial thromboembolic event, ischemic stroke and MI compared to the VTE cohort. VTE cohort had higher all-cause mortality while AF cohort had higher CV mortality. Therefore, VTE and AF patients have different risks in presentations related to arterial thromboembolic events.

Long-term anticoagulation therapy to prevent arterial thromboembolism is a well-established strategy in AF population (2) and a net clinical benefit more than 5 years with DOACs is still evident (14). On the other hand, long-term anticoagulation was not recommended in VTE population due to uncertain net clinical benefit in the era of vitamin K antagonists (VKA, e.g., warfarin) (15). However, some studies have shown that extended treatment with DOAC for 6-15 months resulted in less recurrent VTE events than no treatment, and had less bleeding events compared to VKA (29–32). Of note, extended low-dose aspirin in VTE patients for up to 4 years results in a significant reduction in the rate of major vascular events, with improved net clinical benefit in the ASPIRE study (33). Moreover, our study showed that anticoagulant use had a significant impact on the association between AF/VTE and individual arterial thromboembolic events and all-cause and cardiovascular mortality (Table 3). Lifelong anticoagulation is indicated in AF patients with high CHA_2_DS_2_-VASc score (≥2). The differential manifestations of thromboembolism sequelae and mortality between AF and VTE cohorts merit further investigation of an extended period or lifelong anticoagulation in VTE patients and validation in other ethnic population.

Our study has several limitations. First, we could not clearly identify the prevalence of provoked and unprovoked VTE in our VTE cohort. Several observational studies have reported that unprovoked VTE does not contribute to the same risk as provoked VTE in terms of clinical outcomes, including arterial thromboembolic events. However, a 20-year national observational cohort study reported no significant differences in arterial CV events between provoked and unprovoked VTE (23). In addition, the distinction between provoked/unprovoked PE is no longer supported by the 2019 ESC guidelines for the diagnosis and management of acute pulmonary embolism (34). Furthermore, clinical presentations/manifestations and laboratory data were not available in NHIRD and such information might affect the outcomes of VTE, especially those with PE (34). In order to reduce the bias, we excluded those died during hospitalization and within 3 months after discharge. Second, differentiating subtypes of AF (paroxysmal, sustained) cannot be performed because this information was not available in our national database. Although the incidence of ischemic stroke is considered to be generally lower in paroxysmal AF patients than in patients with sustained AF (35), it should not affect the outcomes between AF and VTE in such a large volume study. Third, the duration of anticoagulation therapy was different between AF vs. VTE cohorts (2, 34). We also did not compare the outcomes between AF and VTE cohorts in individual different scenarios with different durations of anticoagulation therapy. Furthermore, although there were different frequencies of anticoagulants and antiplatelets between AF and VTE cohorts before propensity matching (Table 1), our conclusion was based on the results derived from the study population after propensity matching (Tables 1, 2). Therefore, the unbalanced prescription of anticoagulants and antiplatelets between AF and VTE cohorts before propensity matching should not influence our main results derived from the study population after propensity matching. Fourth, VTE cohort had a higher incidence of lower extremity thromboembolic events than AF cohort. We could not completely exclude the possibility of more image studies performed in the VTE cohort to reveal a higher incidence rate of lower extremity thromboembolic events. Finally, propensity score matching was used to reduce the potential confounding variables in this study. However, there were potential unknown variables in the study population for matching and comparison.

## Conclusion

Patients with AF had higher risks of arterial thromboembolic events (ischemic stroke and MI) compared to patients with VTE, despite having risk factors in common. The VTE cohort had higher risks of all-cause mortality and ECATE, particularly lower extremity events, compared to AF patients. The differential manifestations of thromboembolism sequelae and mortality between AF and VTE patients merit further investigation of an extended period or lifelong anticoagulation in VTE patients.

## Data Availability Statement

The data underlying this study is from the Taiwan's National Health Insurance Research Database (NHIRD), which has been transferred to the Health and Welfare Data Science Center (HWDC). Interested researchers can obtain the data through formal application to the HWDC, Department of Statistics, Ministry of Health and Welfare, Taiwan (http://dep.mohw.gov.tw/DOS/np-2497-113.html). Requests to access these datasets should be directed to (http://dep.mohw.gov.tw/DOS/np-2497-113.html).

## Ethics Statement

The study was approved by the Institutional Review Board of Chang Gung Memorial Hospital: IRB number: 201900915B1. Written informed consent for participation was not required for this study in accordance with the national legislation and the institutional requirements.

## Author Contributions

Y-SL, M-SL, GL, and M-CC: study concept and design. VW, Y-LC, and J-JC: acquisition of data. Y-SL, M-SL, and P-HC: analysis and interpretation of data. Y-SL, GL, and M-CC: manuscript draft. GL and M-CC: critical revision of the manuscript for important intellectual content. All authors reviewed the manuscript and completed final approval.

## Conflict of Interest

GL is a Consultant for Bayer/Janssen, BMS/Pfizer, Medtronic, Boehringer Ingelheim, Novartis, Verseon, and Daiichi-Sankyo. Speaker for Bayer, BMS/Pfizer, Medtronic, Boehringer Ingelheim, and Daiichi-Sankyo. The remaining authors declare that the research was conducted in the absence of any commercial or financial relationships that could be construed as a potential conflict of interest.

## Publisher's Note

All claims expressed in this article are solely those of the authors and do not necessarily represent those of their affiliated organizations, or those of the publisher, the editors and the reviewers. Any product that may be evaluated in this article, or claim that may be made by its manufacturer, is not guaranteed or endorsed by the publisher.
