# Supplementary material for: Differential Presentations of Arterial Thromboembolic Events Between Venous Thromboembolism and Atrial Fibrillation Patients
Source: Front Cardiovasc Med. 2021 Dec 6;8:775564. doi: 10.3389/fcvm.2021.775564 (PMC8685417; doi:10.3389/fcvm.2021.775564)

**Supplemental Table 1**. Baseline characteristics of the patients diagnosed with DVT-only or AF

|  | Before propensity matching | | |  | After propensity matching | | |
| --- | --- | --- | --- | --- | --- | --- | --- |
|  | DVT-only  (n = 29,512) | AF  (n = 314,861) | STD |  | DVT-only  (n = 24,330) | AF  (n = 24,330) | STD |
| Age (years) | 64.0 ± 16.3 | 71.2 ± 13.5 | -0.48 |  | 66.0 ± 15.5 | 67.0 ± 14.6 | -0.07 |
| Age group |  |  |  |  |  |  |  |
| < 65 years | 13,916 (47.2) | 89,486 (28.4) | 0.39 |  | 10,251 (42.1) | 9,638 (39.6) | 0.05 |
| 65-74 years | 6,773 (22.9) | 82,459 (26.2) | -0.08 |  | 5,913 (24.3) | 6,103 (25.1) | -0.02 |
| ≥ 75 years | 8,823 (29.9) | 142,916 (45.4) | -0.32 |  | 8,166 (33.6) | 8,589 (35.3) | -0.04 |
| Male sex | 13,890 (47.1) | 174,954 (55.6) | -0.17 |  | 12,022 (49.4) | 12,318 (50.6) | -0.02 |
| Comorbid conditions |  |  |  |  |  |  |  |
| Hypertension | 15,060 (51.0) | 191,993 (61.0) | -0.20 |  | 13,367 (54.9) | 14,037 (57.7) | -0.06 |
| Diabetes mellitus | 7,884 (26.7) | 75,303 (23.9) | 0.06 |  | 6,690 (27.5) | 6,876 (28.3) | -0.02 |
| Ischemic heart disease | 5,431 (18.4) | 110,828 (35.2) | -0.39 |  | 5,099 (21.0) | 5,588 (23.0) | -0.05 |
| Dyslipidemia | 5,152 (17.5) | 52,525 (16.7) | 0.02 |  | 4,466 (18.4) | 4,716 (19.4) | -0.03 |
| Gout | 3,014 (10.2) | 31,540 (10.0) | 0.01 |  | 2,638 (10.8) | 2,724 (11.2) | -0.01 |
| COPD | 3,298 (11.2) | 58,210 (18.5) | -0.21 |  | 2,955 (12.1) | 2,968 (12.2) | <0.01 |
| Peripheral artery disease | 2,301 (7.8) | 11,400 (3.6) | 0.18 |  | 1,739 (7.1) | 1,795 (7.4) | -0.01 |
| Chronic kidney disease | 5,272 (17.9) | 45,300 (14.4) | 0.09 |  | 4,398 (18.1) | 4,511 (18.5) | -0.01 |
| Dialysis | 1,011 (3.4) | 8,190 (2.6) | 0.05 |  | 876 (3.6) | 901 (3.7) | -0.01 |
| Cancer | 7,042 (23.9) | 19,901 (6.3) | 0.51 |  | 4,175 (17.2) | 3,947 (16.2) | 0.03 |
| Auto-immune disease | 545 (1.8) | 1,891 (0.6) | 0.11 |  | 310 (1.3) | 283 (1.2) | 0.01 |
| Hepatitis C virus infection | 627 (2.1) | 4,841 (1.5) | 0.04 |  | 491 (2.0) | 466 (1.9) | 0.01 |
| Paralysis | 2,449 (8.3) | 22,491 (7.1) | 0.04 |  | 2,114 (8.7) | 2,272 (9.3) | -0.02 |
| Osteoporosis | 2,557 (8.7) | 19,662 (6.2) | 0.09 |  | 2,068 (8.5) | 1,940 (8.0) | 0.02 |
| Charlson Comorbidity Index score | 3.1 ± 3.1 | 2.0 ± 2.0 | 0.43 |  | 2.7 ± 2.8 | 2.8 ± 2.7 | -0.01 |
| History of disease |  |  |  |  |  |  |  |
| Prior any stroke | 4,020 (13.6) | 46,969 (14.9) | -0.04 |  | 3,635 (14.9) | 3,887 (16.0) | -0.03 |
| Prior ischemic stroke or systemic thromboembolism | 3,999 (13.6) | 45,757 (14.5) | -0.03 |  | 3,593 (14.8) | 3,905 (16.1) | -0.04 |
| Old MI | 857 (2.9) | 13,564 (4.3) | -0.08 |  | 794 (3.3) | 917 (3.8) | -0.03 |
| Heart failure admission | 2,323 (7.9) | 40,121 (12.7) | -0.16 |  | 2,175 (8.9) | 2,417 (9.9) | -0.03 |
| Antithrombotic therapy within 3 months after index date |  |  |  |  |  |  |  |
| None | 6,921 (23.5) | 119,550 (38.0) | -0.32 |  | 6,833 (28.1) | 6,375 (26.2) | 0.04 |
| Antiplatelet | 2,337 (7.9) | 148,174 (47.1) | -0.98 |  | 2,335 (9.6) | 2,563 (10.5) | -0.03 |
| Anticoagulant | 20,254 (68.6) | 47,137 (15.0) | 1.30 |  | 15,162 (62.3) | 15,392 (63.3) | -0.02 |
| Medication |  |  |  |  |  |  |  |
| ACEi or ARB | 7,403 (25.1) | 137,957 (43.8) | -0.40 |  | 6,974 (28.7) | 7,721 (31.7) | -0.07 |
| Beta blocker | 5,559 (18.8) | 115,262 (36.6) | -0.41 |  | 5,275 (21.7) | 5,773 (23.7) | -0.05 |
| DCCB | 6,844 (23.2) | 85,295 (27.1) | -0.09 |  | 6,042 (24.8) | 6,333 (26.0) | -0.03 |
| Diuretic | 7,099 (24.1) | 80,723 (25.6) | -0.04 |  | 5,828 (24.0) | 6,080 (25.0) | -0.02 |
| Metformin | 2,945 (10.0) | 35,237 (11.2) | -0.04 |  | 2,549 (10.5) | 2,707 (11.1) | -0.02 |
| TZD | 510 (1.7) | 4,942 (1.6) | 0.01 |  | 416 (1.7) | 441 (1.8) | -0.01 |
| DPP4i | 527 (1.8) | 5,783 (1.8) | 0.00 |  | 486 (2.0) | 555 (2.3) | -0.02 |
| Insulin | 1,426 (4.8) | 10,125 (3.2) | 0.08 |  | 1,156 (4.8) | 1,210 (5.0) | -0.01 |
| Estrogen | 759 (2.6) | 4,105 (1.3) | 0.09 |  | 487 (2.0) | 451 (1.9) | 0.01 |
| Antidepressants | 2,653 (9.0) | 20,564 (6.5) | 0.09 |  | 2,085 (8.6) | 2,166 (8.9) | -0.01 |
| Statin | 2,952 (10.0) | 39,303 (12.5) | -0.08 |  | 2,713 (11.2) | 3,082 (12.7) | -0.05 |
| Digoxin | 607 (2.1) | 73,147 (23.2) | -0.67 |  | 607 (2.5) | 705 (2.9) | -0.02 |
| Follow up year | 3.8 ± 3.5 | 4.2 ± 3.4 | -0.10 |  | 3.87 ± 3.5 | 3.93 ± 3.5 | -0.02 |

Abbreviations: VTE, venous thromboembolism; AF, atrial fibrillation; STD, standardized difference; COPD, chronic obstructive pulmonary disease; ACEi, angiotensin converting enzyme inhibitor; ARB, angiotensin receptor blocker; DCCB, dihydropyrinde calcium channel blocker; OHA, oral hypoglycemic agent; TZD, thiazolidinedione; DDP4i, dipeptidyl peptidase 4 inhibitor;

Data were presented as frequency (percentage) or mean ± standard deviation.

**Supplemental Table 2**. Baseline characteristics of the patients diagnosed with PE-only or AF

|  | Before propensity matching | | |  | After propensity matching | | |
| --- | --- | --- | --- | --- | --- | --- | --- |
|  | PE-only  (n = 9,143) | AF  (n = 314,861) | STD |  | PE-only  (n = 8,879) | AF  (n = 8,879) | STD |
| Age (years) | 65.8 ± 16.5 | 71.2 ± 13.5 | -0.36 |  | 66.2 ± 16.3 | 66.6 ± 15.4 | -0.02 |
| Age group |  |  |  |  |  |  |  |
| < 65 years | 3,741 (40.9) | 89,486 (28.4) | 0.26 |  | 3,548 (40.0) | 3,502 (39.4) | 0.01 |
| 65-74 years | 2,196 (24.0) | 82,459 (26.2) | -0.05 |  | 2,160 (24.3) | 2,132 (24.0) | 0.01 |
| ≥ 75 years | 3,206 (35.1) | 142,916 (45.4) | -0.21 |  | 3,171 (35.7) | 3,245 (36.5) | -0.02 |
| Male sex | 4,114 (45.0) | 174,954 (55.6) | -0.21 |  | 4,052 (45.6) | 4,047 (45.6) | <0.01 |
| Comorbid conditions |  |  |  |  |  |  |  |
| Hypertension | 5,028 (55.0) | 191,993 (61.0) | -0.12 |  | 4,941 (55.6) | 5,012 (56.4) | -0.02 |
| Diabetes mellitus | 2,373 (26.0) | 75,303 (23.9) | 0.05 |  | 2,320 (26.1) | 2,382 (26.8) | -0.02 |
| Ischemic heart disease | 2,852 (31.2) | 110,828 (35.2) | -0.09 |  | 2,800 (31.5) | 2,918 (32.9) | -0.03 |
| Dyslipidemia | 1,715 (18.8) | 52,525 (16.7) | 0.05 |  | 1,674 (18.9) | 1,728 (19.5) | -0.02 |
| Gout | 849 (9.3) | 31,540 (10.0) | -0.02 |  | 835 (9.4) | 860 (9.7) | -0.01 |
| COPD | 1,964 (21.5) | 58,210 (18.5) | 0.07 |  | 1,905 (21.5) | 1,910 (21.5) | <0.01 |
| Peripheral artery disease | 378 (4.1) | 11,400 (3.6) | 0.03 |  | 367 (4.1) | 393 (4.4) | -0.01 |
| Chronic kidney disease | 1,587 (17.4) | 45,300 (14.4) | 0.08 |  | 1,534 (17.3) | 1,598 (18.0) | -0.02 |
| Dialysis | 179 (2.0) | 8,190 (2.6) | -0.04 |  | 177 (2.0) | 189 (2.1) | -0.01 |
| Cancer | 1,443 (15.8) | 19,901 (6.3) | 0.31 |  | 1,322 (14.9) | 1,326 (14.9) | <0.01 |
| Auto-immune disease | 214 (2.3) | 1,891 (0.6) | 0.14 |  | 180 (2.0) | 163 (1.8) | 0.01 |
| Hepatitis C virus infection | 168 (1.8) | 4,841 (1.5) | 0.02 |  | 160 (1.8) | 179 (2.0) | -0.02 |
| Paralysis | 634 (6.9) | 22,491 (7.1) | -0.01 |  | 626 (7.1) | 634 (7.1) | <0.01 |
| Osteoporosis | 806 (8.8) | 19,662 (6.2) | 0.10 |  | 783 (8.8) | 759 (8.5) | 0.01 |
| Charlson Comorbidity Index score | 2.9 ± 2.8 | 2.0 ± 2.0 | 0.37 |  | 2.8 ± 2.8 | 2.9 ± 2.7 | -0.01 |
| History of disease |  |  |  |  |  |  |  |
| Prior any stroke | 1,323 (14.5) | 46,969 (14.9) | -0.01 |  | 1,308 (14.7) | 1,389 (15.6) | -0.03 |
| Prior ischemic stroke or systemic thromboembolism | 1,272 (13.9) | 45,757 (14.5) | -0.02 |  | 1,258 (14.2) | 1,333 (15.0) | -0.02 |
| Old MI | 328 (3.6) | 13,564 (4.3) | -0.04 |  | 325 (3.7) | 361 (4.1) | -0.02 |
| Heart failure admission | 1,133 (12.4) | 40,121 (12.7) | -0.01 |  | 1,106 (12.5) | 1,186 (13.4) | -0.03 |
| Antithrombotic therapy within 3 months after index date |  |  |  |  |  |  |  |
| None | 2,369 (25.9) | 119,550 (38.0) | -0.26 |  | 2,368 (26.7) | 2273 (25.6) | 0.03 |
| Antiplatelet | 790 (8.6) | 148,174 (47.1) | -0.95 |  | 790 (8.9) | 875 (9.9) | -0.03 |
| Anticoagulant | 5,984 (65.4) | 47,137 (15.0) | 1.20 |  | 5,721 (64.4) | 5,731 (64.5) | <0.01 |
| Medication |  |  |  |  |  |  |  |
| ACEi or ARB | 2,711 (29.7) | 137,957 (43.8) | -0.30 |  | 2,685 (30.2) | 2,720 (30.6) | -0.01 |
| Beta blocker | 1,992 (21.8) | 115,262 (36.6) | -0.33 |  | 1,983 (22.3) | 1,966 (22.1) | <0.01 |
| DCCB | 2,255 (24.7) | 85,295 (27.1) | -0.06 |  | 2,217 (25.0) | 2,228 (25.1) | <0.01 |
| Diuretic | 2,143 (23.4) | 80,723 (25.6) | -0.05 |  | 2,076 (23.4) | 2,190 (24.7) | -0.03 |
| Metformin | 894 (9.8) | 35,237 (11.2) | -0.05 |  | 875 (9.9) | 873 (9.8) | <0.01 |
| TZD | 120 (1.3) | 4,942 (1.6) | -0.02 |  | 120 (1.4) | 111 (1.3) | 0.01 |
| DPP4i | 201 (2.2) | 5,783 (1.8) | 0.03 |  | 196 (2.2) | 206 (2.3) | -0.01 |
| Insulin | 392 (4.3) | 10,125 (3.2) | 0.06 |  | 384 (4.3) | 396 (4.5) | -0.01 |
| Estrogen | 148 (1.6) | 4,105 (1.3) | 0.03 |  | 140 (1.6) | 133 (1.5) | 0.01 |
| Antidepressants | 863 (9.4) | 20,564 (6.5) | 0.11 |  | 825 (9.3) | 866 (9.8) | -0.02 |
| Statin | 1,021 (11.2) | 39,303 (12.5) | -0.04 |  | 1,014 (11.4) | 1,076 (12.1) | -0.02 |
| Digoxin | 391 (4.3) | 73,147 (23.2) | -0.57 |  | 390 (4.4) | 418 (4.7) | -0.02 |
| Follow up year | 3.5 ± 3.4 | 4.2 ± 3.4 | -0.21 |  | 3.5 ± 3.4 | 3.7 ± 3.3 | -0.05 |

Abbreviations: VTE, venous thromboembolism; AF, atrial fibrillation; STD, standardized difference; COPD, chronic obstructive pulmonary disease; ACEi, angiotensin converting enzyme inhibitor; ARB, angiotensin receptor blocker; DCCB, dihydropyrinde calcium channel blocker; TZD, thiazolidinedione; DDP4i, dipeptidyl peptidase 4 inhibitor;

Data were presented as frequency (percentage) or mean ± standard deviation.

**Supplemental Table 3**. Baseline characteristics of the patients diagnosed with PE-only or DVT-only

|  | Before propensity matching | | |  | After propensity matching | | |
| --- | --- | --- | --- | --- | --- | --- | --- |
|  | PE-only  (n = 9,143) | DVT-only  (n = 29,512) | STD |  | PE-only  (n = 8,725) | DVT-only  (n = 8,725) | STD |
| Age (years) | 65.8 ± 16.5 | 64.0 ± 16.3 | 0.11 |  | 65.9 ± 16.5 | 65.9 ± 16.5 | <0.01 |
| Age group |  |  |  |  |  |  |  |
| < 65 years | 3,741 (40.9) | 13,916 (47.2) | -0.13 |  | 3,547 (40.7) | 3,538 (40.6) | <0.01 |
| 65-74 years | 2,196 (24.0) | 6,773 (22.9) | 0.03 |  | 2,087 (23.9) | 2,058 (23.6) | 0.01 |
| ≥ 75 years | 3,206 (35.1) | 8,823 (29.9) | 0.11 |  | 3,091 (35.4) | 3,129 (35.9) | -0.01 |
| Male sex | 4,114 (45.0) | 13,890 (47.1) | -0.04 |  | 3,987 (45.7) | 3,916 (44.9) | 0.02 |
| Comorbid conditions |  |  |  |  |  |  |  |
| Hypertension | 5,028 (55.0) | 15,060 (51.0) | 0.08 |  | 4,817 (55.2) | 4,846 (55.5) | -0.01 |
| Diabetes mellitus | 2,373 (26.0) | 7,884 (26.7) | -0.02 |  | 2,280 (26.1) | 2,213 (25.4) | 0.02 |
| Ischemic heart disease | 2,852 (31.2) | 5,431 (18.4) | 0.30 |  | 2,699 (30.9) | 2,676 (30.7) | 0.01 |
| Dyslipidemia | 1,715 (18.8) | 5,152 (17.5) | 0.03 |  | 1,638 (18.8) | 1,637 (18.8) | <0.01 |
| Gout | 849 (9.3) | 3,014 (10.2) | -0.03 |  | 809 (9.3) | 777 (8.9) | 0.01 |
| COPD | 1,964 (21.5) | 3,298 (11.2) | 0.28 |  | 1,833 (21.0) | 1,768 (20.3) | 0.02 |
| Peripheral artery disease | 378 (4.1) | 2,301 (7.8) | -0.16 |  | 371 (4.3) | 336 (3.9) | 0.02 |
| Chronic kidney disease | 1,587 (17.4) | 5,272 (17.9) | -0.01 |  | 1,525 (17.5) | 1,486 (17.0) | 0.01 |
| Dialysis | 179 (2.0) | 1,011 (3.4) | -0.09 |  | 177 (2.0) | 156 (1.8) | 0.02 |
| Cancer | 1,443 (15.8) | 7,042 (23.9) | -0.20 |  | 1,422 (16.3) | 1,398 (16.0) | 0.01 |
| Auto-immune disease | 214 (2.3) | 545 (1.8) | 0.03 |  | 187 (2.1) | 186 (2.1) | <0.01 |
| Hepatitis C virus infection | 168 (1.8) | 627 (2.1) | -0.02 |  | 163 (1.9) | 176 (2.0) | -0.01 |
| Paralysis | 634 (6.9) | 2,449 (8.3) | -0.05 |  | 620 (7.1) | 635 (7.3) | -0.01 |
| Osteoporosis | 806 (8.8) | 2,557 (8.7) | 0.01 |  | 775 (8.9) | 797 (9.1) | -0.01 |
| Charlson Comorbidity Index score | 2.9 ± 2.8 | 3.1 ± 3.1 | -0.07 |  | 2.92 ± 2.9 | 2.90 ± 2.9 | 0.01 |
| History of disease |  |  |  |  |  |  |  |
| Prior any stroke | 1,323 (14.5) | 4,020 (13.6) | 0.02 |  | 1,279 (14.7) | 1,317 (15.1) | -0.01 |
| Prior ischemic stroke or systemic thromboembolism | 1,272 (13.9) | 3,999 (13.6) | 0.01 |  | 1,233 (14.1) | 1,237 (14.2) | <0.01 |
| Old MI | 328 (3.6) | 857 (2.9) | 0.04 |  | 317 (3.6) | 331 (3.8) | -0.01 |
| Heart failure admision | 1,133 (12.4) | 2,323 (7.9) | 0.15 |  | 1,029 (11.8) | 1,019 (11.7) | <0.01 |
| Antithrombotic therapy within 3 months after index date |  |  |  |  |  |  |  |
| None | 2,369 (25.9) | 6,921 (23.5) | 0.06 |  | 2,290 (26.2) | 2,275 (26.1) | <0.01 |
| Antiplatelet | 790 (8.6) | 2,337 (7.9) | 0.03 |  | 768 (8.8) | 735 (8.4) | 0.01 |
| Anticoagulant | 5,984 (65.4) | 20,254 (68.6) | -0.07 |  | 5,667 (65.0) | 5,715 (65.5) | -0.01 |
| Medication |  |  |  |  |  |  |  |
| ACEi or ARB | 2,711 (29.7) | 7,403 (25.1) | 0.10 |  | 2,572 (29.5) | 2,592 (29.7) | -0.01 |
| Beta blocker | 1,992 (21.8) | 5,559 (18.8) | 0.07 |  | 1,912 (21.9) | 1,960 (22.5) | -0.01 |
| DCCB | 2,255 (24.7) | 6,844 (23.2) | 0.03 |  | 2,154 (24.7) | 2,257 (25.9) | -0.03 |
| Diuretic | 2,143 (23.4) | 7,099 (24.1) | -0.01 |  | 2,000 (22.9) | 2,039 (23.4) | -0.01 |
| Metformin | 894 (9.8) | 2,945 (10.0) | -0.01 |  | 863 (9.9) | 845 (9.7) | 0.01 |
| TZD | 120 (1.3) | 510 (1.7) | -0.03 |  | 115 (1.3) | 104 (1.2) | 0.01 |
| DPP4i | 201 (2.2) | 527 (1.8) | 0.03 |  | 194 (2.2) | 196 (2.2) | <0.01 |
| Insulin | 392 (4.3) | 1,426 (4.8) | -0.03 |  | 382 (4.4) | 340 (3.9) | 0.02 |
| Estrogen | 148 (1.6) | 759 (2.6) | -0.07 |  | 144 (1.7) | 146 (1.7) | <0.01 |
| Antidepressants | 863 (9.4) | 2,653 (9.0) | 0.02 |  | 837 (9.6) | 843 (9.7) | <0.01 |
| Statin | 1,021 (11.2) | 2,952 (10.0) | 0.04 |  | 990 (11.3) | 1,002 (11.5) | <0.01 |
| Digoxin | 391 (4.3) | 607 (2.1) | 0.13 |  | 338 (3.9) | 341 (3.9) | <0.01 |
| Follow up year | 3.5 ± 3.4 | 3.8 ± 3.5 | -0.10 |  | 3.47 ± 3.4 | 3.48 ± 3.3 | <0.01 |

Abbreviations: VTE, venous thromboembolism; AF, atrial fibrillation; STD, standardized difference; COPD, chronic obstructive pulmonary disease; ACEi, angiotensin converting enzyme inhibitor; ARB, angiotensin receptor blocker; DCCB, dihydropyrinde calcium channel blocker; TZD, thiazolidinedione; DDP4i, dipeptidyl peptidase 4 inhibitor;

Data were presented as frequency (percentage) or mean ± standard deviation.

**Supplemental Table 4.** The causes of death of patients with VTE versus those with AF in the propensity score matched cohort

|  | Before propensity matching | |  | After propensity matching | |
| --- | --- | --- | --- | --- | --- |
| Death reasons | VTE  (*n* = 14,188) | AF  (*n* = 12,345) |  | VTE  (*n* = 10,572) | AF  (*n* = 9,574) |
| CV | 5,958 (42.0) | 6,376 (51.6)* |  | 5,958 (56.4) | 6,376 (66.6)* |
| Non-CV death |  |  |  |  |  |
| Cancer related death | 1,449 (10.2) | 820 (6.6)* |  | 1,449 (13.7) | 820 (8.6)* |
| Infection other than pneumonia | 742 (5.2) | 541 (4.4)* |  | 742 (7.0) | 541 (5.7)* |
| Pneumonia | 357 (2.5) | 342 (2.8) |  | 357 (3.4) | 342 (3.6) |
| GI bleeding | 369 (2.6) | 244 (2.0)* |  | 369 (3.5) | 244 (2.5)* |
| Trauma | 127 (0.9) | 94 (0.8) |  | 127 (1.2) | 94 (1.0) |
| Other | 1570 (11) | 1157 (9.4)* |  | 1570 (14.8) | 1157 (12.0)* |
| Unknown | 3,616 (25.5)* | 2,771 (22.4) |  | - | - |

Abbreviations: VTE, venous thromboembolism; AF, atrial fibrillation; CV, cardiovascular; GI, gastrointestinal;

** P* <0.05.

**Supplemental Table 5.** Follow-up outcomes in patients with DVT-only cohort versus AF cohort

|  | Before propensity matching | |  | After propensity matching | | | |
| --- | --- | --- | --- | --- | --- | --- | --- |
| Outcome | DVT-only  (n = 29,512) | AF  (*n* = 314,861) |  | DVT-only  (*n* = 24,330) | AF  (*n* = 24,330) | HR or SHR of DVT-only  (95% CI) | *P* |
| Arterial thromboembolic events | 3,704 (12.6) | 61,684 (19.6) |  | 3,264 (13.4) | 4,870 (20.0) | 0.62 (0.59–0.65) | <.001 |
| Ischemic stroke | 2,146 (7.3) | 47,867 (15.2) |  | 1,923 (7.9) | 3,892 (16.0) | 0.45 (0.43–0.48) | <.001 |
| Extracranial arterial thromboembolism | 1,412 (4.8) | 10,026 (3.2) |  | 1,196 (4.9) | 897 (3.7) | 1.31 (1.20–1.43) | <.001 |
| Lower extremity thromboembolism | 1,224 (4.1) | 8,361 (2.7) |  | 1,040 (4.3) | 742 (3.0) | 1.37 (1.25–1.51) | <.001 |
| Non-lower extremity thromboembolism | 244 (0.8) | 2,329 (0.7) |  | 206 (0.847) | 208 (0.85) | 0.96 (0.79–1.17) | 0.704 |
| Myocardial infarction | 481 (1.6) | 9,718 (3.1) |  | 438 (1.8) | 556 (2.3) | 0.76 (0.67–0.86) | <.001 |
| Secondary outcomes |  |  |  |  |  |  |  |
| All-cause mortality | 13,179 (44.7) | 135,551 (43.1) |  | 10,481 (43.1) | 9,383 (38.6) | 1.14 (1.11–1.17) | <.001 |
| Cardiovascular death | 5,025 (17.0) | 73,344 (23.3) |  | 4,172 (17.2) | 4,784 (19.7) | 0.89 (0.85–0.92) | <.001 |
| Non-cardiovascular death | 8,154 (27.6) | 62,207 (19.8) |  | 6,309 (25.9) | 4,599 (18.9) | 1.40 (1.34–1.45) | <.001 |

Abbreviations: DVT, deep vein thrombosis; AF, atrial fibrillation; HR, hazard ratio; SHR, subdistribution hazard ratio; CI, confidence interval;

Data were presented as frequency (percentage).

**Supplemental Table 6.** Follow-up outcomes in patients with PE-only cohort versus AF cohort

|  | Before propensity matching | |  | After propensity matching | | | |
| --- | --- | --- | --- | --- | --- | --- | --- |
| Outcome | PE-only  (*n* = 9,143) | AF  (*n* = 314,861) |  | PE-only  (*n* = 8,879) | AF  (*n* = 8,879) | HR or SHR of PE-only  (95% CI) | *P* |
| Arterial thromboembolic events | 900 (9.8) | 61,684 (19.6) |  | 884 (10.0) | 1,560 (17.6) | 0.52 (0.48–0.56) | <.001 |
| Ischemic stroke | 573 (6.3) | 47,867 (15.2) |  | 564 (6.4) | 1,263 (14.2) | 0.41 (0.37–0.45) | <.001 |
| Extracranial arterial thromboembolism | 228 (2.5) | 10,026 (3.2) |  | 223 (2.5) | 228 (2.6) | 0.94 (0.79–1.14) | 0.539 |
| Lower extremity thromboembolism | 149 (1.6) | 8,361 (2.7) |  | 147 (1.7) | 179 (2.0) | 0.79 (0.64–0.98) | 0.033 |
| Non-lower extremity thromboembolism | 90 (1.0) | 2,329 (0.7) |  | 86 (1.0) | 65 (0.7) | 1.28 (0.93–1.76) | 0.128 |
| Myocardial infarction | 175 (1.9) | 9,718 (3.1) |  | 171 (1.9) | 188 (2.1) | 0.87 (0.71–1.08) | 0.204 |
| Secondary outcomes |  |  |  |  |  |  |  |
| All-cause mortality | 4,049 (44.3) | 135,551 (43.1) |  | 3,904 (44.0) | 3,246 (36.6) | 1.26 (1.20–1.32) | <.001 |
| Cardiovascular death | 1,897 (20.8) | 73,344 (23.3) |  | 1,838 (20.7) | 1,688 (19.0) | 1.14 (1.07–1.22) | <.001 |
| Non-cardiovascular death | 2,152 (23.5) | 62,207 (19.8) |  | 2,066 (23.3) | 1,558 (17.6) | 1.39 (1.30–1.48) | <.001 |

Abbreviations: PE, pulmonary embolism; AF, atrial fibrillation; HR, hazard ratio; SHR, subdistribution hazard ratio; CI, confidence interval.

Data were presented as frequency (percentage).

**Supplemental Table 7.** Follow-up outcomes in patients with PE-only versus those with DVT-only

|  | Before propensity matching | |  | After propensity matching | | | |
| --- | --- | --- | --- | --- | --- | --- | --- |
| Outcome | PE-only  (*n* = 9,143) | DVT-only  (*n* = 29,512) |  | PE-only  (*n* = 8,725) | DVT-only  (*n* = 8,725) | HR or SHR of PE-only  (95% CI) | *P* |
| Arterial thromboembolic events | 900 (9.8) | 3,704 (12.6) |  | 869 (10.0) | 1,053 (12.1) | 0.80 (0.73–0.88) | <.001 |
| Ischemic stroke | 573 (6.3) | 2,146 (7.3) |  | 556 (6.4) | 659 (7.6) | 0.82 (0.74–0.92) | 0.001 |
| Extracranial arterial thromboembolism | 228 (2.5) | 1,412 (4.8) |  | 221 (2.5) | 313 (3.6) | 0.69 (0.58–0.82) | <.001 |
| Lower extremity thromboembolism | 149 (1.6) | 1,224 (4.1) |  | 145 (1.7) | 267 (3.1) | 0.53 (0.43–0.65) | <.001 |
| Non-lower extremity thromboembolism | 90 (1.0) | 244 (0.8) |  | 86 (1.0) | 52 (0.6) | 1.64 (1.16–2.31) | 0.005 |
| Myocardial infarction | 175 (1.9) | 481 (1.6) |  | 167 (1.9) | 166 (1.9) | 0.99 (0.80–1.22) | 0.910 |
| Associated outcomes |  |  |  |  |  |  |  |
| All-cause mortality | 4,049 (44.3) | 13,179 (44.7) |  | 3,865 (44.3) | 3,622 (41.5) | 1.08 (1.03–1.13) | 0.001 |
| Cardiovascular death | 1,897 (20.8) | 5,025 (17.0) |  | 1,786 (20.5) | 1,417 (16.2) | 1.28 (1.19–1.37) | <.001 |
| Non-cardiovascular death | 2,152 (23.5) | 8,154 (27.6) |  | 2,079 (23.8) | 2,205 (25.3) | 0.96 (0.90–1.02) | 0.137 |

Abbreviations: PE, pulmonary embolism; DVT, deep vein thrombosis; HR, hazard ratio; SHR, subdistribution hazard ratio; CI, confidence interval;

Data were presented as frequency (percentage).

**Supplemental Table 8.** Summarized comparisons of outcomes among AF, DVT-only and PE-only cohorts

| Outcome | AF versus VTE | AF, DVT-only and PE-only |
| --- | --- | --- |
| Arterial thromboembolic events | AF > VTE | AF > DVT > PE |
| Ischemic stroke | AF > VTE | AF > DVT > PE |
| Extracranial arterial thromboembolism | VTE > AF | DVT > PE, DVT > AF, PE = AF |
| Lower extremity thromboembolism | VTE > AF | DVT > AF > PE |
| Non-lower extremity thromboembolism | AF = VTE | PE > DVT, DVT = AF, PE = AF |
| Myocardial infarction | AF > VTE | AF > DVT, AF ≈ PE, DVT = PE |
| Mortality |  |  |
| All-cause mortality | VTE > AF | PE > DVT > AF |
| Cardiovascular death | AF > VTE | PE > AF > DVT |
| Non-cardiovascular death | VTE > AF | PE = DVT > AF |

Abbreviations: AF, atrial fibrillation; VTE, venous thromboembolism; DVT, deep vein thrombosis; PE, pulmonary embolism

**Supplemental Figure 1.** The cumulative incidence rate of arterial thromboembolic event (A), ischemic stroke (B), extracranial arterial thromboembolic events (C), cardiovascular death (D) and all-cause mortality (E) after propensity score matching between patients with deep vein thrombosis (DVT) alone and patients with atrial fibrillation (AF).


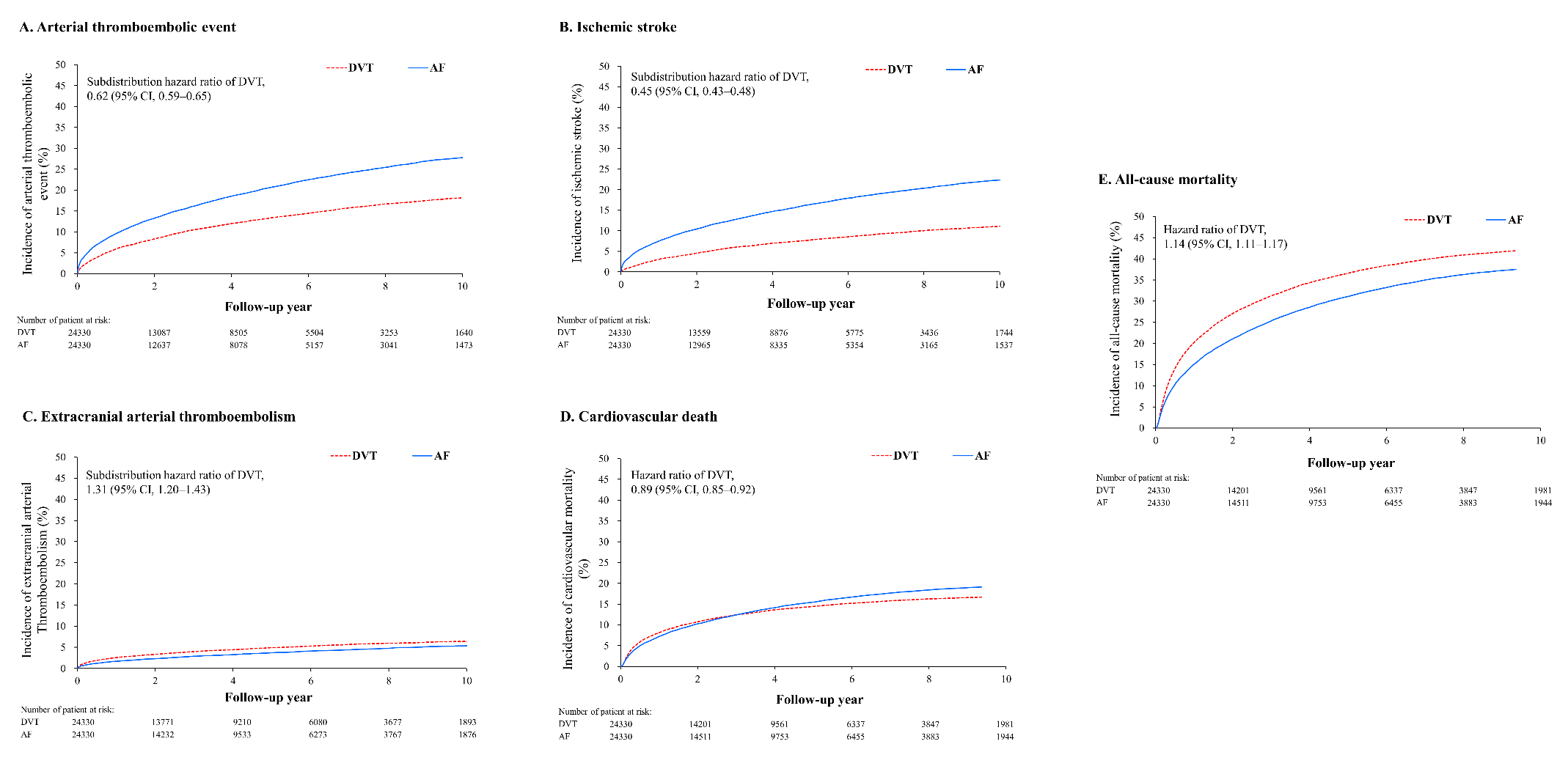


**Supplemental Figure 2.** The cumulative incidence rate of arterial thromboembolic event (A), ischemic stroke (B), extracranial arterial thromboembolic events (C), cardiovascular death (D) and all-cause mortality (E) after propensity score matching between patients with pulmonary embolism (PE) alone and patients with atrial fibrillation (AF).


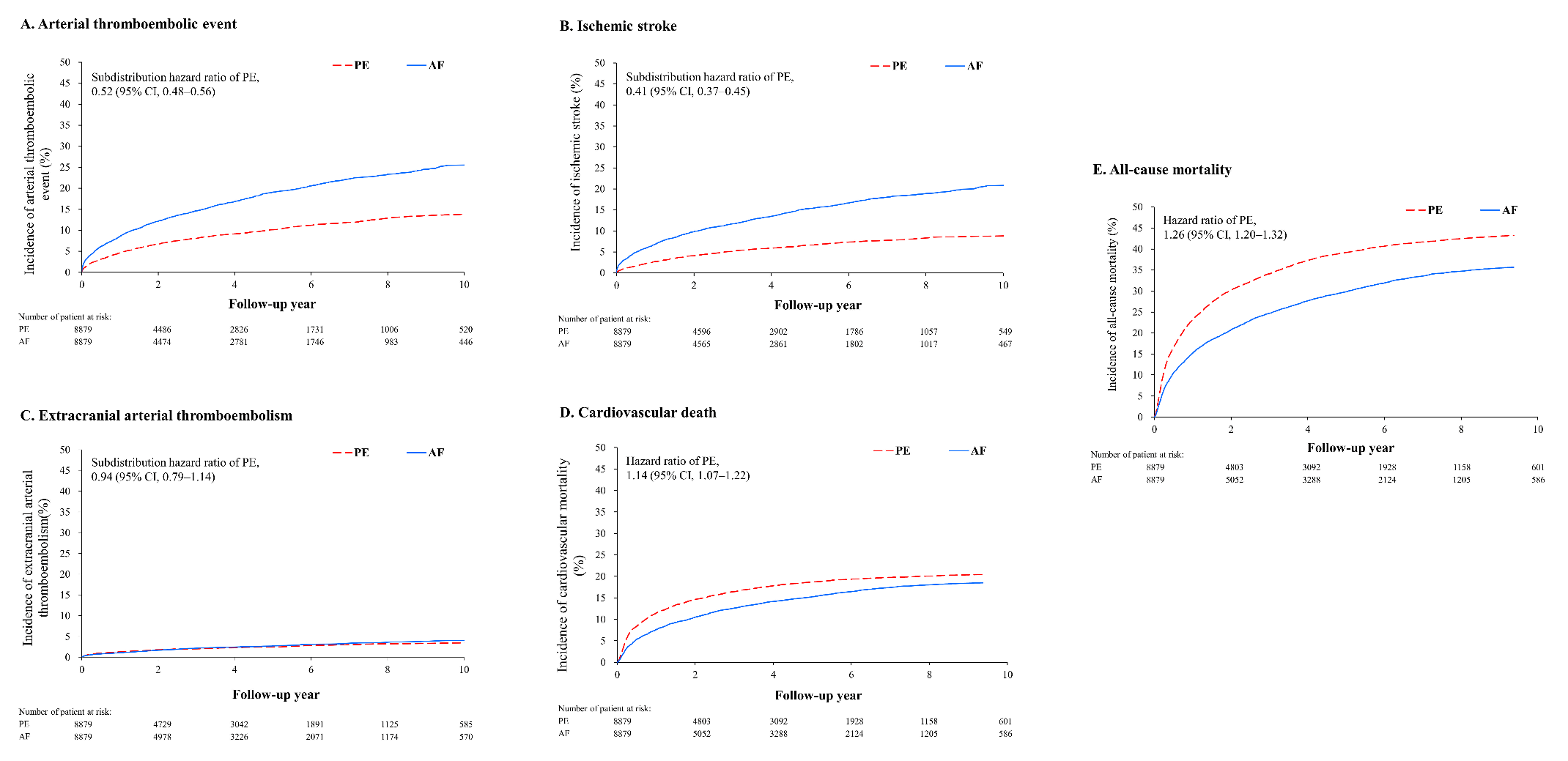

Supplement: Supplementary file 1 [file Data_Sheet_1.docx]
